# Understanding Knowledge and Behaviors Related to CoViD–19 Epidemic in Italian Undergraduate Students: The EPICO Study

**DOI:** 10.3390/ijerph17103481

**Published:** 2020-05-16

**Authors:** Francesca Gallè, Elita Anna Sabella, Giovanna Da Molin, Osvalda De Giglio, Giuseppina Caggiano, Valeria Di Onofrio, Stefano Ferracuti, Maria Teresa Montagna, Giorgio Liguori, Giovanni Battista Orsi, Christian Napoli

**Affiliations:** 1 Department of Movement Sciences and Wellbeing, University of Naples “Parthenope”, Via Medina n. 40, 80133 Naples, Italy; giorgio.liguori@uniparthenope.it; 2 Inter-University Research Centre “Population, Environment and Health”, University of Bari Aldo Moro, Piazza Umberto I, 1, 70121 Bari, Italy; elita.sabella@uniba.it (E.A.S.); giovanna.damolin@uniba.it (G.D.M.); 3 Department of Biomedical Science and Human Oncology, University of Bari Aldo Moro, Piazza G. Cesare 11, 70124 Bari, Italy; osvalda.degiglio@uniba.it (O.D.G.); giuseppina.caggiano@uniba.it (G.C.); mariateresa.montagna@uniba.it (M.T.M.); 4 Department of Sciences and Technologies, University of Naples “Parthenope”, Business District, Block C4, 80143 Naples, Italy; valeria.dionofrio@uniparthenope.it; 5 Department of Human Neurosciences, “Sapienza” University of Rome, Piazzale Aldo Moro 5, 00185 Rome, Italy; stefano.ferracuti@uniroma1.it; 6 Department of Public Health and Infectious Diseases, “Sapienza” University of Rome, Piazzale Aldo Moro 5, 00185 Rome, Italy; giovanni.orsi@uniroma1.it; 7 Department of Medical Surgical Sciences and Translational Medicine, “Sapienza” University of Rome, Via di Grottarossa 1035/1039, 00189 Rome, Italy; christian.napoli@uniroma1.it

**Keywords:** CoViD–19, knowledge, behaviors, quarantine, undergraduates

## Abstract

*Background:* On February 2020, the novel coronavirus (2019−nCoV) epidemic began in Italy. In order to contain the spread of the virus, the Italian government adopted emergency measures nationwide, including closure of schools and universities, workplaces and subsequently lockdown. This survey was carried out among Italian undergraduates to explore their level of knowledge about the epidemic and the behaviors they adopted during the lockdown. *Methods:* An electronic questionnaire was administered to the students attending three Italian universities. *Results:* A good level of knowledge about the epidemic and its control was registered in the sample, mainly among students attending life sciences degree courses. The majority of the students did not modify their diet and smoking habits, while a great part of the sample reported a decrease in physical activity (PA). *Conclusions:* Students from life sciences courses showed a higher awareness regarding the infection and the control measures. The lockdown caused an important reduction of PA. Preventive interventions should transform the restrictive measures also as an opportunity to improve lifestyle.

## 1. Introduction

The epidemic caused by the novel Coronavirus (2019−nCoV) started in China at the end of the 2019 and has spread rapidly worldwide [1]. With regard to our continent, before the 2009 influenza A(H1N1) pandemic, most European Union (EU) Member States had developed preparedness plans in order to respond in a timely manner to an eventual pandemic. Many of these plans involve explicit or implicit planning assumptions on what can be expected during a pandemic and on how a pandemic virus might behave [2]. This is particularly important in a world characterized by global movements of population [3]. Unfortunately, the Italian pandemic plan was not enough to stop the start of the epidemic also in Italy. Therefore, at the end of February 2020, since the first case of coronavirus−19 disease (CoViD–19) was registered in Northern Italy, the epidemic involved gradually the whole Italian country [4]. At 19 April 2020, Italy has had 175,925 confirmed cases, and 23,227 deaths [1].

In order to contain the epidemic, the Presidency of the Italian Council of Ministries gave the responsibility of the emergency management to its Civil Protection Department and adopted a series of control measures such as information campaigns, closure of schools and universities, workplaces and subsequently lockdown. These measures were applied first in the northern regions and later in the whole Italian territory [5,6]. The last decree limits the movement of individuals in the whole Italian national territory unless strictly motivated (in written form) by reasons of work or health. Shops must stay closed but those selling essentials, such as supermarkets or pharmacies need to ensure a distance of at least 1 m between customers. Schools, museums, cinemas, theatres, and any other social, recreational, or cultural center must stay closed. Any gathering in public spaces is forbidden, including sporting events and funerals. At the same time, in order to minimize the possible side effects of the lockdown on health, the Italian Ministry of Health issued a series of recommendations targeted at four rules for maintaining a healthy lifestyle: correct diet, daily physical activity (PA), reduce alcohol consumption and no smoking [7]. These measures are without precedent [8]. As for previous pandemic events, the social, economic and psychological impact of the epidemic is enormous [9]. It is demonstrated that, in order to contain the epidemic and to limit its consequences, people’s adherence to the recommended measures is essential, and this is influenced by knowledge, attitudes, and practices towards the disease [10]. During the Severe Acute Respiratory Syndrome (SARS) outbreak which happened in 2003, knowledge and attitudes towards infectious diseases were associated with fear, which were able to hinder the efforts to prevent the spread of the disease [11]. Furthermore, also in the course of the present epidemic, a higher knowledge regarding CoViD–19 was found to be associated with a lower likelihood of negative attitudes and potentially dangerous practices in the Chinese population [12]. With regard to Italy, the level of knowledge about the disease and the lifestyle adopted by the Italian general population, during the current epidemic, has not been investigated so far. In order to fill this gap, it is necessary to investigate the aspects of knowledge and its eventual consequences on lifestyle behaviors.

Therefore, this study was conducted in a sample of Italian undergraduate students during the COVID−19 epidemic in order to: (i) evaluate the level of knowledge about the 2019-nCoV, its spread and the control measures adopted; (ii) analyze health-related behaviors during lockdown, in order to estimate its possible impact on personal habits; (iii) understand if the study field may influence the level of knowledge and lifestyle habits during the pandemic.

## 2. Materials and Methods

Given that the objective of the study was analyzing data collected at one given point in time, across a sample population (undergraduate students) similar in most variables except for study field, a cross-sectional study was designed. This choice was also based on previous experiences conducted during the COVID−19 pandemic [12,13].

### 2.1. Setting and Participants

The “Survey on knowledge and behaviors of undergraduates during the EPIdemic of COronavirus−19” (EPICO study) took place across the two last weeks of March 2020, involving three Italian universities: The University of Rome La Sapienza, the University of Naples Parthenope and the University of Bari Aldo Moro. The estimated whole population was 166,703 students. During that period, with the total number of confirmed cases increasing from about 26,000 to 77,000, the CoViD–19 epidemic was growing in the whole Italian territory, and no classroom lessons were allowed due to the emergency measures established on 8 March [5]. Therefore, universities encouraged the teachers to provide their lessons via the internet. All the students attending web courses in the three universities were invited, during lessons, to voluntarily participate in the study by filling in a web-based questionnaire. The use of phone/web direct interviews was not considered, in order to enroll higher numbers of participants.

A sample of at least 384 enrolled individuals would have been required to investigate the selected variables in the students’ population examined. The sample was calculated by a sample size calculator, based on the reference population of undergraduate students of the three universities and assuming a response proportion of 50%, a 95% confidence level and a 5% margin error, as previously reported [13].

The investigation was performed in accordance with the World Medical Association Declaration of Helsinki and did not include any experiments involving human or biological human samples, nor research on identifiable human data. Therefore, the protocol was approved by the Scientific and Ethical committee of the Inter University Research Centre “Population, environment and health” (CIRPAS), with the number 1603_2020.

### 2.2. Questionnaire

With respect to the recommendation regarding the avoidance of close contacts and touch precautions during the outbreak, we designed an online questionnaire, in the Italian language, to perform the survey. This choice was also adopted in similar studies, demonstrating its strength in enrolling a large sample during a critical period [12,13].

The questionnaire had been previously tested in a pilot-study (data not published). The measure of how comprehensible the questions were considered was evaluated in a group of 185 undergraduate students who were not included in the larger study sample. The 185 students were asked to assign a rating to each question on a 7-point scale (Q: Does the following sentence make sense to you? 1: not meaningful; 7: very meaningful); a mean score >5 per question was considered as the cut off for acceptability. For this purpose, the original questionnaire was modified: aside from the questions belonging to the standard questionnaire (SQQ), 10 additional questions (AQ) reporting grammatical and semantic errors (e.g., the use of the verb to be in place of to have, the use of general words such as “things” in place of specific words etc.) were included, in order to guarantee answer variability. SQQ reported a mean score for each question > 6 (very close to the maximum score); AQ reported a mean score for each question <3. These data confirmed that the content of the questionnaire was clear to the readers. In the same pilot sample, the reliability index for SQQ was assessed using Cronbach’s alpha (internal consistency coefficient) [14,15], for both the pilot and original study. The alpha values achieved were 0.79 and 0.81, respectively, showing a satisfactory level of reliability also considering that the questionnaire did not include items of high difficulty [16].

The questionnaire was composed of three sections.

The first section included questions regarding demographic information (gender, age, university, degree type, year of the course attended). These items were designed based on a previous study in the same student population [17] and the opinions of a panel of experts composed of one demographer and one epidemiologist.

The second section, aimed at assessing the undergraduates’ knowledge about CoViD–19 and its control measures, was based on a framework from a review on the disease [18] and on the statements of the national laws [6]. The validity of the items included in this section was established by a panel of experts including two epidemiologists, one physician expert in public health and one psychologist. During this process, selected literature was thoroughly screened to identify all the useful references; experts were also given the opportunity to produce a specific indication as expert opinions. Moreover, to further support the questionnaire validity by confirming our starting hypothesis, the 185 students of the pilot study were divided into two groups, one of which was presumed to have a higher knowledge on CoViD–19, since following a life science degree. Knowledge of the two groups were compared; students attending a life sciences degree scored consistently higher than the other group (*p* < 0.001).

Cronbach’s alpha for this section was 0.72 and 0.78 for the pilot and original study, respectively.

Students were asked:

1. To express an opinion regarding their own level of knowledge on the new epidemic (excellent, good, average, low);
2. To judge if the new epidemic may be considered similar/non-similar to that of the seasonal flu;
3. To refer if the CoViD–19 may be treated with the available antibiotics;
4. To report if they know someone who was affected by the CoVid−2019;
5. To identify the cause of the new epidemic as a virus, a bacterium, or a parasite;
6. To report the main transmission route (air/water/food/blood transfusion/sexual intercourse);
7. To report the most effective protective measures (handwashing/mask wearing/use of gloves/protective glasses/fruit and vegetables washing/use of condom/disinfection of surfaces/avoid close contacts);
8. To reply about the availability of an effective drug for CoViD–19 treatment;
9. To reply about the availability of a vaccine for its prevention (yes/no);
10. To identify the target population of CoViD–19 (only older people/only children/only adults/all);
11. To identify the area of the Italian territory involved in the epidemic (Northern regions/Center regions/South regions/all);
12. To identify the reason for the lockdown (reduce the severity of the disease/decrease the number of infected people/enhance the immune status of the population/reduce the burden of patients in hospitals);
13. To report the categories who underwent diagnostic tests for 2019-nCoV detection (only patients with acute symptoms/patients with acute symptoms and their contacts/all the people who live in “red-zones”/only health personnel);
14. To report which institution was managing the emergency in Italy (Ministry of Health/National Health Institute/Council of Ministers/Civil Protection Department/Regions);
15. To refer the number of people infected at the time of the survey (5,000/50,000/5,000,000).

The third part of the questionnaire was focused on the lifestyle behaviors adopted by the students during the lockdown with respect to pre-epidemic conditions. According to the recommendations of the Italian Ministry of Health about healthy lifestyles during the CoViD–19 epidemic, three main behaviors were investigated [7]. The validity of the items included in this section was established by a panel of experts including one epidemiologist, one biologist expert in human nutrition, one biologist expert in movement sciences and one psychologist. Cronbach’s alpha for this section was 0.74 and 0.79 for the pilot and original study, respectively.

The students were asked to report their habits regarding:

1. Current diet (eating as before/eating less or better than before/eating more or worse than before);
2. Smoking (no smoking before nor currently/no smoking before but now yes/smoking before and now/smoking before and not now);
3. PA (increased/decreased/active as before/inactive as before).

### 2.3. Statistical Analyses

A descriptive analysis was performed considering demographic characteristics and answers provided by the sample as a whole and grouped on the basis of type of degree course – life science/others, assuming that life sciences students were more acquainted with biology and health protection principles.

Continuous outcomes were expressed as mean values ± standard deviation (SD). The Student’s t-test was used to compare mean values between the two educational groups.

Data regarding conditions and behaviors were reported as the number and percentage of respondents; these variables were compared between the two groups using the chi-squared test.

A logistic regression analysis was performed considering the level of knowledge showed by participants about the epidemic and its control as the outcome. The dependent variable was built calculating the median value of the total number of correct answers given by students and attributing the value 0 if this sum was lower than the median value, and 1 otherwise. Age, gender, educational field and knowing someone who was affected by CoViD–19 were assumed as independent variables.

Data were analyzed using IBM SPSS version 26 for Windows (IBM Corp., Armonk, NY, USA).

## 3. Results

On the whole student population of the three Universities (*n* = 166,703), a total of 2125 students completely fulfilled the questionnaire (response rate: 1.3). Overall, 1047 students (49.3%) attended a degree course in the area of life sciences, while the remaining 1078 (50.7%) attended a degree course in other areas.

The main characteristics of participants grouped by educational area are reported in Table 1.

Table 2 shows the answers provided by participants regarding their knowledge about the disease and the epidemic. The majority of the sample, and mainly life sciences students, considered their level of knowledge about the epidemic good. The current epidemic was considered different from that of the seasonal flu by about the three quarters of the sample, without differences between groups.

More than 80% of the participants recognized that antibiotics were not effective for treating CoViD–19, but this percentage was significantly higher among life sciences students than in the other group. A similar situation was registered regarding the cause of CoViD–19: more than 93% of the total sample identified correctly a virus, but the two groups showed significant differences. Air was identified as the main route of transmission by 89% of the sample, without differences between groups. Avoiding close contacts, washing hands and wearing facial masks were considered the most protective measures to avoid the disease by the whole sample and by both groups; a higher percentage of life sciences students identified the right proposed measures.

About 70% of the sample suggested that a specific drug to treat CoViD–19 was not available, while 90.1% of the sample was aware that a vaccine was still lacking, without differences between groups. The proportion of the sample who knew that CoViD–19 may affect all people independently by age was 90.2%, but this value was higher in life sciences students, as well as the percentage of participants who reported that the epidemic interested the whole country rather than some geographical areas.

The decrease of cases was identified as the main objective of lockdown by 86.4% of the sample; the two groups differed mainly in the identification of the burden and severity reduction.

Patients with acute symptoms and their contacts were identified as the categories who underwent diagnostics tests for CoViD–19 by about half of the sample, with significant differences between groups.

The Ministry of Health was identified as the institution which manages the CoViD–19 emergency by the majority of the sample, while the Civil Protection Department was referred to by 27.5% of the sample, without differences between groups.

87.5% of the sample reported a number of infected people close to 50,000 in Italy; the correct answer was more frequently chosen by life sciences students.

Table 3 shows the answers of students from the two educational fields regarding their lifestyles during the lockdown. As for dietary habits, the majority of life sciences students declared that they did not change their habits, while the greatest part of the others reported an improvement in their diet. The majority of the sample did not smoke neither before nor during the lockdown, while 34% maintained their smoking habit. Life sciences students showed a lower proportion of smokers than the others, while these showed a higher proportion of quitters. PA levels decreased in about half of the sample; however, part of both groups was able to maintain or increase the usual PA practice. In particular, compared to the other group, the life sciences group showed a higher proportion of students who continued to exercise at the same level, while a greater part of the others increased their PA levels, even if they also showed a higher number of individuals who tended to maintain their previous inactivity. All the behavioral differences between groups were significant.

Table 4 shows the results of the logistic regression performed considering the number of correct answers given by students regarding the epidemic and its control as the outcome. Female gender was positively associated with a number of correct answers equal to or higher than the median value (12), while belonging to educational fields different from life sciences and not knowing someone affected by CoViD–19 seemed to be inversely related with a better knowledge.

## 4. Discussion

To the best of our knowledge, this is the first study aimed to assess the level of knowledge of Italian undergraduates about CoViD–19 and its control measures and to explore their behaviors during the epidemic. The results of the investigation showed a good level of knowledge in the majority of the sample, with differences between students attending life sciences degree courses and those attending other courses. These findings are consistent with the results of other studies recently performed among healthcare students [13,19]. Even if we did not investigated the information sources used by students, it should be considered that the lockdown has given students a greater opportunity to watch television and surf the web while staying at home and, in this way, have been able to improve their level of knowledge [20]. In this period, in fact, mass media have given continuous and timely updates on the pandemic evolution. Mass media have a significant influence both on the knowledge and attitudes of people [21], as well as on their risk perception [22]. In fact, measures to control the world epidemic include not only identifying new organisms, developing vaccines, and initiating appropriate therapies, but also adequately informing the public about risks and precautions [23]. In this context, mass communication media are valuable resources for efficiently communicating risk information to the public; nevertheless, the role of the mass media in risk communication, within health and more generally, is often debated. In a survey of 2005, Bergeron and Sanchez investigated the general knowledge of SARS in Canadian undergraduate students and concluded that the Canadian media communicated contradictory messages and generated confusion in the population [24]. Therefore, an extensive collaboration among public health departments and media outlets is essential to deliver health information to all the sectors of the society [25,26]. The lower levels of knowledge registered among students from degree courses different from those related to life sciences testifies that the educational field may also play a role in determining the comprehension of the control measures recommended, which is related to the adoption of appropriate measures in order to limit the spread of the disease [12]. A lower level of knowledge was also associated with attending courses different from life science in the regression analysis. Considering these differences, health education programs aimed at improving CoViD–19 knowledge in unaware groups may be helpful to support people in maintaining safe practices, especially in the perspective of the forthcoming end to the lockdown.

Furthermore, female gender was positively associated with a better knowledge of the disease and the epidemic control, while not knowing someone affected by CoViD–19 was associated with a lower number of correct answers. With regard to these aspects, it was previously shown that women are more knowledgeable and have appropriate practices towards CoViD–19 [12]. Furthermore, it is demonstrated that having relatives or acquaintances infected with CoViD–19 is a risk factor for increasing the anxiety of college students, and, therefore, to increase their knowledge about the disease and its control measure [27].

As for the examined lifestyles, it seems that the Ministerial recommendations were generally followed because the majority of the students did not modify their diet and smoking habits, but a decrease of PA level was reported in almost half of the sample. This represents an important issue, considering that in the context of the current epidemic the interest for the potential role of PA as an immune function adjuvant to reduce the risk of communicable diseases has increased appreciably. Epidemiological evidences have demonstrated a dose-response relationship between PA performed before infection and a reduction in the incidence, duration, or severity of acute respiratory tract infections [28]. The role of modifiable lifestyle factors like diet and PA in maintaining health and wellness are fundamental. Additionally, in times of crisis, the benefits of empowering people to actively preserve their own health should be underlined [29].

In the comparison between the two groups, life sciences students showed more stability in their previous diet, smoking and PA behaviors than the other group. This is in accordance with a previous investigation carried out in a sample from the same students’ population, which showed an association between attending life science courses and healthy behaviors [17]. However, in the present study, students attending other courses reported higher improvements in dietary, smoking and PA habits than life sciences students did. This suggests that the lockdown has offered to the other group a greater opportunity to improve behaviors. Probably their willingness to adopt a better lifestyle is normally hindered by other factors that did not emerge during this investigation. The identification of these factors may be useful to support these individuals in improving their health-related behaviors.

The authors are aware of some limitations of the study. First, lifestyles were not investigated in depth, in order to avoid an excessive length of the questionnaire. This could have hidden important information related to behaviors as well as other sociodemographic variables that were not collected. Furthermore, this study was aimed at exploring knowledge and lifestyles of a sample of undergraduate students from three Italian universities, who represent a specific population group and are not representative of the whole population of young adults in Italy. Moreover, the sample showed a higher female component: this probably reflects the different compliance to the investigation of the two genders, as reported in a similar investigation during the CoViD–19 pandemic [12,13]. Therefore, the present study can be considered just preliminary research. Due to the limitation in representativeness, further studies are needed to deepen the investigation in this sub population. Nevertheless, during a critical period of emergency, it may help to better address the future public information campaigns earlier and highlight the need to emphasize the importance of adopting a healthy lifestyle.

## 5. Conclusions

The findings of this investigation testify an acceptable level of undergraduate students’ knowledge regarding the epidemic and the control measures adopted. Life sciences students showed a higher awareness than the others towards the infection mechanisms and the procedures to prevent its spread. As for lifestyles, the majority of the students did not modify their diet and smoking habits but reported a decrease in PA level.

Although there are certain limits, this study may early help, during the current emergency period, to better address the future public information campaigns regarding CoViD–19 prevention and highlights the need to further emphasize the importance of adopting a healthy lifestyle, especially exercise, during the lockdown.

## Figures and Tables

**Table 1:** Characteristics of the sample.

Variable	Whole Sample *n* = 2125	Life Sciences *n* = 1047	Other *n* = 1078	*p* Value
Age *Mean ± SD*	22.5 ± 0.08	22.7 ± 3.43	22.3 ± 4.11	0.01 ^1^
Gender *n* (%)				
Male	791 (37.2)	411 (39.3)	380 (35.3)	0.06 ^2^
Female	1334 (62.8)	636 (60.7)	698 (64.7)	
Knowing Someone Affected by CoViD–19 *n* (%)	363 (17.1)	192 (18.3)	171 (15.9)	0.13 ^2^

^1^*t* test; ^2^ chi-squared test.

**Table 2:** Answers provided by students regarding the CoViD–19 pandemic.

Questions *n* of Respondents (%)	Whole Sample *n* = 2125	Life Sciences *n* = 1047	Other *n* = 1078	*p* Value
Your Knowledge about Covid−19 Epidemic is				
Excellent	230 (10.8)	117 (11.2)	113 (10.5)	0.04
Good	1211 (57)	610 (58.3)	601 (55.8)	
Medium	593 (27.9)	288 (27.5)	305 (28.3)	
Low	91 (4.3)	32 (3.1)	59 (5.5)	
The New Epidemic may be Considered Similar to that of the Seasonal Flu?				
Yes	523 (24.6)	252 (24.1)	271 (25.1)	0.56
No	1602 (75.4)	795 (75.9)	807 (74.9)	
Is it Possible to Treat Covid−19 with Antibiotics?				
Yes	410 (19.3)	156 (14.9)	254 (23.6)	0.01
No	1715 (80.7)	891 (85.1)	824 (76.4)	
The Cause of Covid−19 is a				
Virus	1978 (93.1)	1016 (97.0)	962 (89.2)	0.00
Bacterium	125 (5.9)	21 (2)	104 (9.6)	
Parasite	22 (1)	10 (1)	12 (1.1)	
* The Main Transmission Route of the Disease is				
Air	1891 (89)	942 (90)	949 (88)	0.33
Water	301 (14.2)	161 (15.4)	140 (13)	
Food	298 (14)	153 (14.6)	145 (13.5)	
Blood Transfusion	413 (19.4)	205 (19.6)	208 (19.3)	
Sexual Intercourse	473 (22.3)	228 (21.8)	245 (22.7)	
* The most Effective Protective Measure is				
Handwashing	1854 (87.2)	933 (89.1)	921 (85.4)	0.00
Facial Masks	1848 (87.0)	912 (87.1)	936 (86.8)	
Gloves	1646 (77.5)	815 (77.8)	831 (77.1)	
Protective Glasses	698 (32.8)	422 (40.3)	276 (25.6)	
Fruit and Vegetables Washing	529 (24.9)	284 (27.1)	245 (22.7)	
Use of Condom	188 (8.8)	105 (10.0)	83 (7.7)	
Disinfection of Surfaces	1557 (73.3)	785 (75.0)	772 (71.6)	
Avoid Close Contacts	1860 (87.5)	917 (87.6)	943 (87.5)	
Is there a Drug to Treat Covid−19?				
Yes	643 (30.3)	310 (29.6)	333 (30.9)	0.52
No	1482 (69.7)	737 (70.4)	745 (69.1)	
Is there a Vaccine to Prevent Covid−19?				
Yes	210 (9.9)	99 (9.5)	111 (10.3)	0.52
No	1915 (90.1)	948 (90.5)	967 (89.7)	
Which is the Target Population of Covid−19?				
Only Older People	170 (8.0)	70 (6.7)	100 (9.3)	0.01
Only Children	38 (1.8)	12 (1.1)	26 (2.4)	
All	1917 (90.2)	965 (92.2)	952 (88.3)	
Which Area of the Italian Territory has been Involved in the Epidemic?				
Northern Regions	389 (18.3)	173 (16.5)	216 (20)	0.01
Center Regions	30 (1.4)	8 (0.8)	22 (2)	
South Regions	20 (0.9)	7 (0.7)	13 (1.2)	
All	1686 (79.3)	859 (82)	827 (76.7)	
* The Lockdown has been Adopted to:				
Reduce the Severity of the Disease	191 (9.0)	82 (7.8)	109 (10.1)	0.03
Decrease Infected People	1837 (86.4)	911 (87.0)	926 (85.9)	
Enhance People Immune Status	43 (2.0)	20 (1.9)	23 (2.1)	
Reduce the Burden Of Patients	869 (40.9)	450 (43.0)	419 (38.9)	
Which Categories Underwent Diagnostic Tests?				
Patients with Acute Symptoms	319 (15)	146 (13.9)	173 (16.0)	0.02
And their Contacts	1082 (50.9)	548 (52.3)	534 (49.5)	
People who Live in “Red-Zones”	680 (32.0)	323 (30.9)	357 (33.1)	
Only Health Personnel	44 (2.1)	30 (2.9)	14 (1.3)	
Which Institution is Managing the Emergency in Italy?				
Ministry of Health	682 (32.1)	347 (33.1)	335 (31.1)	0.18
Superior Health Institute	420 (19.8)	215 (20.5)	205 (19.0)	
Council of Ministers	388 (18.3)	178 (17.0)	210 (19.5)	
Civil Protection Department	585 (27.5)	277 (26.5)	308 (28.6)	
Regions	50 (2.4)	30 (2.9)	20 (1.9)	
How many Italian People have been Affected by CoViD–19?				
5000	194 (9.1)	89 (8.5)	105 (9.7)	0.04
50,000	1859 (87.5)	932 (89.0)	927 (86.0)	
5,000,000	72 (3.4)	26 (2.5)	46 (4.3)	

* Multiple answers were allowed.

**Table 3:** Answers provided by students from the two groups about their behaviors during the lockdown.

*n* (%)	Whole Sample *n* = 2125	Life Sciences *n* = 1047	Other *n* = 1078	*p* Value
Diet				
Same as before	890 (41.9)	487 (46.5)	403 (37.4)	0.00
Less or better than before	871 (41.0)	368 (35.1)	503 (46.7)	
More or worse than before	364 (17.1)	192 (18.3)	172 (16.0)	
Smoking				
Not before nor currently	1239 (58.3)	640 (61.1)	599 (55.6)	0.04
Not before but now yes	42 (2.0)	22 (2.1)	20 (1.8)	
Yes before and now	723 (34.0)	334 (31.9)	389 (36.0)	
Yes before and not now	121 (5.7)	51 (4.9)	70 (6.5)	
Physical Activity				
Decreased	1032 (48.6)	505 (48.2)	527 (48.9)	0.00
Increased	453 (21.3)	212 (20.2)	241 (22.4)	
Active as before	341 (16.0)	209 (20.0)	132 (12.2)	
Inactive as before	299 (14.1)	121 (11.6)	178 (16.5)	

**Table 4:** Results of the logistic regression model built on the number of correct answers as the outcome.

Independent Variable	Number of Correct Answers ≥12 OR (IC95%)
Age ≤21 years ≥22 years	*Reference* 1.11 (0.93−1.32)
Gender Male Female	*Reference* 1.49 (1.25−1.78) **
Educational Area Life Sciences Other	*Reference* 0.75 (0.63−0.89) **
Knowing Someone Affected by CoViD–19 Yes No	*Reference* 0.73 (0.58−0.92) **

** *p* < 0.01.
